# Geography as an Independent Determinant and Immuno‐Inflammatory Correlates of Long‐Term Oral Cancer Survival: A Population‐Based Cohort Study From India

**DOI:** 10.1002/cam4.72078

**Published:** 2026-07-21

**Authors:** Nitika Taneja, Peter Mac Asaga

**Affiliations:** ^1^ Centre for Medicine & Society (Global Health) University Medical Centre Freiburg Freiburg Germany; ^2^ Mahatma Gandhi Institute of Medical Sciences Sevagram Maharashtra India; ^3^ Institute for Infectious Disease Control and Prevention, Faculty of Medicine University Medical Centre Freiburg Freiburg Baden‐Württemberg Germany

**Keywords:** immune–inflammatory biomarkers, India, oral cancer, population‐based registry, survival analysis, urban–rural disparities

## Abstract

**Background:**

India contributes approximately one‐third of global oral cancer cases, with pronounced disparities in outcomes across geographic and socio‐economic strata. While rural–urban survival differences have been documented, limited evidence exists regarding whether geographic residence independently predicts long‐term survival.

**Methods:**

We conducted a retrospective population‐based cohort study using data from the Wardha Population‐Based Cancer Registry, Maharashtra, for oral cancer cases (ICD‐10 C00–C06) diagnosed between 2010 and 2022, with follow‐up through December 2024. Overall survival was analysed using Kaplan–Meier methods and Cox proportional hazards regression. A mechanistic extension incorporated systemic immune–inflammatory biomarkers derived from pretreatment laboratory values, including neutrophil‐to‐lymphocyte ratio, systemic immune–inflammation index, systemic inflammation response index, C‐reactive protein/albumin ratio and CRP–albumin–lymphocyte index.

**Results:**

Among 12,847 registered cancers, 1683 (13.1%) were oral cancers; 66.2% were rural residents. Five‐year survival was 42.1% for rural versus 56.3% for urban residents. Rural residence remained independently associated with mortality after adjustment for age, sex, education and clinical extent (HR 1.34, 95% CI 1.21–1.48). Rural patients demonstrated elevated inflammatory biomarkers and lower CRP–albumin–lymphocyte index. Sequential model adjustment demonstrated attenuation of the rural hazard ratio from 1.41 to 1.22.

**Conclusion:**

Geographic residence independently predicts long‐term oral cancer survival in this population‐based cohort. Systemic immune–inflammatory biomarkers are associated with survival and partially account for rural–urban disparities.

## Graphical abstract

Geographic Disperaties in oral Cancer Survival.

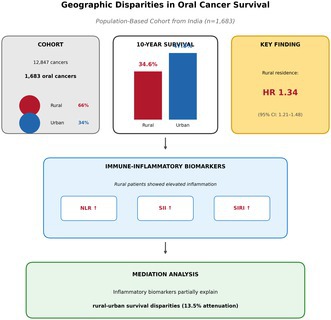

## 1 Introduction

Oral cancer constitutes a major public health burden globally, with India accounting for approximately one‐third of worldwide cases [1, 2]. Population‐based analyses demonstrate that lip and oral cavity cancers rank among the leading causes of cancer‐related mortality in several Indian states, with projections indicating substantial increases in incident cases by 2040 [3, 4]. The epidemiological profile within India exhibits distinctive characteristics: approximately half of oral cancers occur in the buccal mucosa, reflecting culturally specific tobacco quid placement practices, and an estimated 90% of cases are attributable to tobacco consumption [5, 6].

Socio‐demographic factors substantially influence both disease occurrence and survival outcomes. Educational attainment demonstrates an inverse relationship with oral cancer risk, while rural populations face particular vulnerability due to delayed detection, inadequate healthcare infrastructure and economic barriers to timely treatment [7, 8]. These geographic disparities contribute to advanced‐stage presentation, with 5‐year survival rates in India remaining below 35% in many settings [9]. However, most existing evidence derives from hospital‐based cohorts with limited follow‐up duration, and the extent to which geography independently predicts survival after accounting for clinical and demographic factors remains incompletely characterised.

Chronic systemic inflammation and immune dysregulation are increasingly recognised as central determinants of cancer progression and treatment response [10, 11]. In oral squamous cell carcinoma specifically, inflammatory indices derived from routine haematological and biochemical tests have demonstrated prognostic value across multiple populations [12, 13, 14]. These markers may reflect cumulative effects of tobacco exposure, nutritional status, infection burden and delays in care, all of which vary substantially by geographic residence. Integrating immune–inflammatory biomarkers into population‐based survival analyses may therefore strengthen causal inference and provide mechanistic insight into why rural patients experience inferior outcomes.

Wardha district in Maharashtra presents a useful setting for investigating these questions. Earlier work has documented among the highest oral cancer incidence rates for males in the state, yet awareness of risk factors remains suboptimal, with only 30.6% of residents demonstrating knowledge of preventive measures [15, 16]. This study aimed to evaluate long‐term oral cancer survival in a district‐level population‐based cohort over 14 years of accrual and up to 15 years of follow‐up, and to assess whether systemic immune–inflammatory markers contribute to geographic disparities in survival.

## 2 Methods

### 2.1 Study Design and Setting

We conducted a retrospective population‐based cohort study with a nested immune–inflammatory biomarker analysis using data from the Wardha Population‐Based Cancer Registry (PBCR), Maharashtra, India. The study included oral cancer patients diagnosed between 1 January 2010 and 31 December 2022, with survival follow‐up extended to 31 December 2024. Wardha district had an estimated population ranging from 1,309,561 (2012) to 1,420,000 (2022), with approximately 68% residing in rural areas. The registry employed active case finding through municipal councils, Panchayat samitis (district‐level councils) and Gram panchayats (village councils). The study is reported in accordance with the Strengthening the Reporting of Observational Studies in Epidemiology (STROBE) guideline for cohort studies. As an observational cohort study rather than a systematic review, meta‐analysis or scoping review, the PRISMA reporting guideline does not apply to this work.

### 2.2 Study Population

All incident cases of primary oral cancer, defined using International Classification of Diseases 10th Revision (ICD‐10) codes C00–C06, were eligible for inclusion in the survival analysis. For the nested immune–inflammatory analysis, patients were included if pretreatment blood samples were available and collected prior to initiation of any definitive cancer therapy, including surgery, radiotherapy, chemotherapy or immunotherapy. Patients with documented acute infections, inflammatory or autoimmune disorders, or those receiving systemic corticosteroids at the time of blood collection were excluded from the biomarker analysis to minimise confounding of inflammatory indices.

One caveat follows from the design. Because the cohort was assembled retrospectively from routine registry and hospital records, eligibility for the biomarker analysis rested on what had been documented at the point of care rather than on a prespecified research protocol. Acute infection, autoimmune disease and recent corticosteroid use were identified from existing clinical notes, and such notes are not always complete in everyday practice. Some misclassification of inflammatory status is therefore possible, and the exclusion criteria should be read with that limitation in mind.

### 2.3 Laboratory Methods

Peripheral venous blood samples were collected at diagnostic work‐up, prior to oncological intervention. For complete blood count analysis, samples were collected in ethylenediaminetetraacetic acid (EDTA) anticoagulant tubes and analysed within 2 h using an automated haematology analyser calibrated daily according to manufacturer specifications. Absolute neutrophil, lymphocyte, monocyte and platelet counts were recorded. For biochemical analyses, samples were collected in serum separator tubes, allowed to clot and centrifuged at 3000 rpm for 10 min. C‐reactive protein (CRP) was measured using a high‐sensitivity immunoturbidimetric assay, and serum albumin was measured using the bromocresol green method. All assays were performed in laboratories accredited under national quality assurance programmes.

It should be stressed that these values were generated during ordinary clinical work, not under a standardised research protocol. The contributing laboratories operated under national accreditation, yet pre‐analytic conditions such as the interval from venepuncture to analysis, fasting state and sample handling were not controlled prospectively and could not be reconstructed for every patient. The biomarker measurements therefore carry the variability inherent to real‐world laboratory data, and the derived indices should be interpreted with that in view.

### 2.4 Definition of Immune–Inflammatory Biomarkers

Systemic immune–inflammatory biomarkers were calculated using absolute cell counts and biochemical values from pretreatment samples. The neutrophil‐to‐lymphocyte ratio (NLR) was calculated as absolute neutrophil count divided by absolute lymphocyte count. The platelet‐to‐lymphocyte ratio (PLR) was calculated as absolute platelet count divided by absolute lymphocyte count. The lymphocyte‐to‐monocyte ratio (LMR) was calculated as absolute lymphocyte count divided by absolute monocyte count. The systemic immune–inflammation index (SII) was calculated as platelet count × neutrophil count/lymphocyte count. The systemic inflammation response index (SIRI) was calculated as neutrophil count × monocyte count/lymphocyte count. The C‐reactive protein to albumin ratio (CAR) was calculated as serum CRP concentration divided by serum albumin concentration. The CRP–albumin–lymphocyte index (CALLY) was calculated as serum albumin × absolute lymphocyte count/serum CRP concentration. All biomarkers were analysed both as continuous variables and categorised into tertiles based on distribution within the study population.

### 2.5 Clinical and Demographic Variables

Demographic variables included age at diagnosis, sex, place of residence (rural or urban according to administrative classifications) and educational attainment (categorised as illiterate, junior high school, high school or college‐level education). Clinical variables included histological subtype (squamous cell carcinoma, verrucous carcinoma or other/missing) and clinical extent of disease at diagnosis (localised, extended or metastatic) based on clinical examination, imaging findings and pathology reports.

### 2.6 Outcome Ascertainment

The primary outcome was all‐cause mortality. Survival time was calculated from the date of histologically confirmed diagnosis to the date of death from any cause or the date of last follow‐up, whichever occurred first. Patients alive at the end of follow‐up were censored on 31 December 2024. Vital status was ascertained through registry follow‐up procedures, hospital records and linkage with local death registration systems.

### 2.7 Ethical Considerations

The study received approval from the Institutional Ethics Committee of Mahatma Gandhi Institute of Medical Sciences, Sevagram (MGIMS/IEC/INST/2022/MH/0072). The research was conducted in accordance with the Declaration of Helsinki and Indian Council of Medical Research ethical guidelines. Written informed consent was obtained from all participants during registry enrolment for use of their anonymised data in research studies. The study did not involve a clinical trial, and trial registration is therefore not applicable.

### 2.8 Statistical Analysis

Analyses were performed on the full cohort of 1683 oral cancer cases for the descriptive, survival and multivariable modelling, and on the subset of 1260 patients (74.9%) with complete pretreatment laboratory data for the immune–inflammatory biomarker analyses. Continuous variables were screened for distributional form using histograms, normal quantile plots and the Shapiro–Wilk test, and for outliers by inspection of box plots and tabulation of extreme values; implausible biochemical values were checked against source records and retained where confirmed. Normally distributed continuous variables are presented as mean ± standard deviation (SD), skewed continuous variables as median (interquartile range, IQR) and categorical variables as counts and percentages, *n* (%). The immune–inflammatory indices were right‐skewed and were analysed without transformation, both as continuous variables and after categorisation into tertiles; no logarithmic or other transformation was applied. Missing data were handled by complete‐case analysis.

Baseline characteristics were summarised using descriptive statistics. Categorical variables were compared between rural and urban groups using chi‐square tests (two‐sided); continuous variables with skewed distributions were compared using Mann–Whitney *U* tests (two‐sided). Overall survival was estimated using the Kaplan–Meier method, and survival differences were evaluated using the log‐rank test, with the log‐rank test for trend applied to ordered categories such as educational attainment. Cox proportional hazards regression was used to estimate hazard ratios (HR) and 95% confidence intervals (CI) for associations between geographic residence, immune–inflammatory biomarkers and mortality. Multivariable models were constructed sequentially to assess confounding and potential mediation. Model 1 included age and sex. Model 2 additionally included educational attainment. Model 3 added clinical extent of disease. Models 4 and 5 incorporated immune–inflammatory biomarkers individually and in combination to evaluate their independent prognostic value and to assess attenuation of the rural residence hazard ratio. We treated clinical extent as an intermediate on the pathway between residence and death rather than as a simple confounder, since stage at presentation reflects, in part, how late the disease was detected; this positioning is set out further in Section 4. The proportional hazards assumption was assessed using Schoenfeld residuals and graphical methods, specifically scaled Schoenfeld residuals (global and covariate‐specific tests) together with log‐minus‐log survival plots, and was satisfied for all covariates. To evaluate the potential for unmeasured confounding to explain observed associations, *E*‐values were calculated for the primary exposure–outcome relationship.

Biomarker covariates were prespecified; no formal correction for multiple comparisons was applied, and the biomarker associations are accordingly interpreted as hypothesis‐generating where appropriate. Where significance symbols are used in figures and tables, *** denotes *p* < 0.001, ** denotes *p* < 0.01 and * denotes *p* < 0.05 unless otherwise stated. All statistical analyses were performed using Stata version 18 (StataCorp LLC, College Station, Texas, USA). A two‐sided *p*‐value of < 0.05 was considered statistically significant.

## 3 Results

### 3.1 Study Population and Baseline Characteristics

Among 12,847 individuals registered with cancer diagnoses during the study period, 1683 (13.1%) were diagnosed with oral cancer. The male‐to‐female ratio was 2.5:1, with males comprising 71.2% of cases. Mean age at diagnosis was 53.4 years. The 40–49 years age group represented 21.8% of cases, indicating substantial productive life‐years at risk. Geographic analysis revealed 1114 cases (66.2%) among rural residents versus 569 cases (33.8%) among urban dwellers.

Demographic characteristics differed significantly by geographic residence (Table 1). Rural residents were more likely to be male (74.6% vs. 64.5%, *p* < 0.001), younger at presentation (40.9% aged < 50 years vs. 34.1%, *p* = 0.012), and less educated, with 19.8% illiterate compared with 12.8% of urban residents (*p* < 0.001). College‐level education was markedly less common among rural patients (4.8% vs. 19.9%).

**TABLE 1:** Baseline demographic characteristics by geographic residence.

Characteristic	Rural (*n* = 1114)	Urban (*n* = 569)	Total (*n* = 1683)	*p*
Sex				
Male	831 (74.6)	367 (64.5)	1198 (71.2)	< 0.001
Female	283 (25.4)	202 (35.5)	485 (28.8)	
Age group				
< 50 years	456 (40.9)	194 (34.1)	650 (38.6)	0.012
50–59 years	238 (21.4)	120 (21.1)	358 (21.3)	
≥ 60 years	420 (37.7)	255 (44.8)	675 (40.1)	
Educational attainment				
Illiterate	221 (19.8)	73 (12.8)	294 (17.5)	< 0.001
Junior high school	595 (53.4)	252 (44.3)	847 (50.3)	
High school	245 (22.0)	131 (23.0)	376 (22.3)	
College	53 (4.8)	113 (19.9)	166 (9.9)	

*Note:* Data are presented as *n* (%). *p* values from chi‐square tests.

### 3.2 Clinical and Morphological Characteristics

Squamous cell carcinoma accounted for 85.0% of cases (1431/1683), with verrucous carcinoma comprising 7.0% and other or unspecified histologies 8.0% (Table 2). Morphological distribution did not differ significantly by residence (*p* = 0.342). Clinical presentation analysis revealed that 63.8% of cases presented with localised disease, 32.4% with extended involvement and 3.8% with metastatic disease at diagnosis. Rural residents demonstrated a higher proportion with extended disease (33.8% vs. 29.7%, *p* = 0.048), consistent with delayed presentation.

**TABLE 2:** Clinical presentation and morphological characteristics by geographic residence.

Characteristic	Rural (*n* = 1114)	Urban (*n* = 569)	Total (*n* = 1683)	*p*
Histological subtype				
Squamous cell carcinoma	943 (84.7)	488 (85.8)	1431 (85.0)	0.342
Verrucous carcinoma	82 (7.4)	36 (6.3)	118 (7.0)	
Other/missing	89 (7.9)	45 (7.9)	134 (8.0)	
Clinical extent at diagnosis				
Localised	695 (62.4)	378 (66.4)	1073 (63.8)	0.048
Extended	376 (33.8)	169 (29.7)	545 (32.4)	
Metastatic	43 (3.8)	22 (3.9)	65 (3.8)	

*Note:* Data are presented as *n* (%). *p* values from chi‐square tests.

### 3.3 Survival Analysis

**FIGURE 1:**
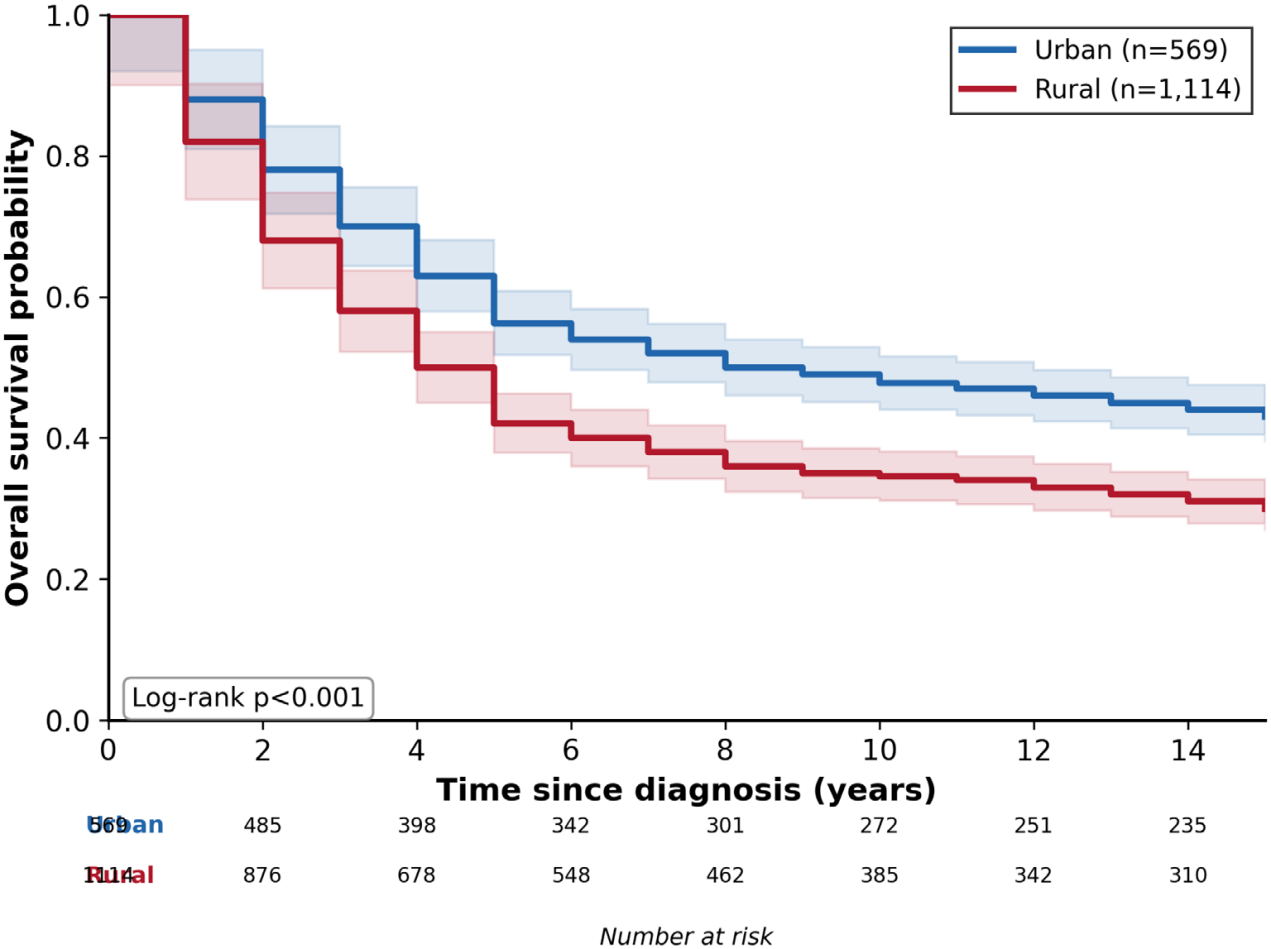
Kaplan–Meier overall survival by geographic residence (urban vs. rural) for the full cohort (*n* = 1683; rural *n* = 1114, urban *n* = 569). Survival probabilities are presented as Kaplan–Meier estimates with 95% confidence intervals, and the two groups were compared using the two‐sided log‐rank test (*p* < 0.001).

Kaplan–Meier survival analysis demonstrated substantial and persistent urban–rural disparities (Figure 1). Five‐year overall survival was 42.1% (95% CI 39.1–45.1) for rural residents compared with 56.3% (95% CI 52.1–60.3) for urban residents (log‐rank *p* < 0.001). Ten‐year survival declined to 34.6% (95% CI 31.7–37.5) and 47.8% (95% CI 43.6–51.9), respectively. The 13.2 percentage‐point difference in 10‐year survival represents substantial excess mortality in rural populations.

**FIGURE 2:**
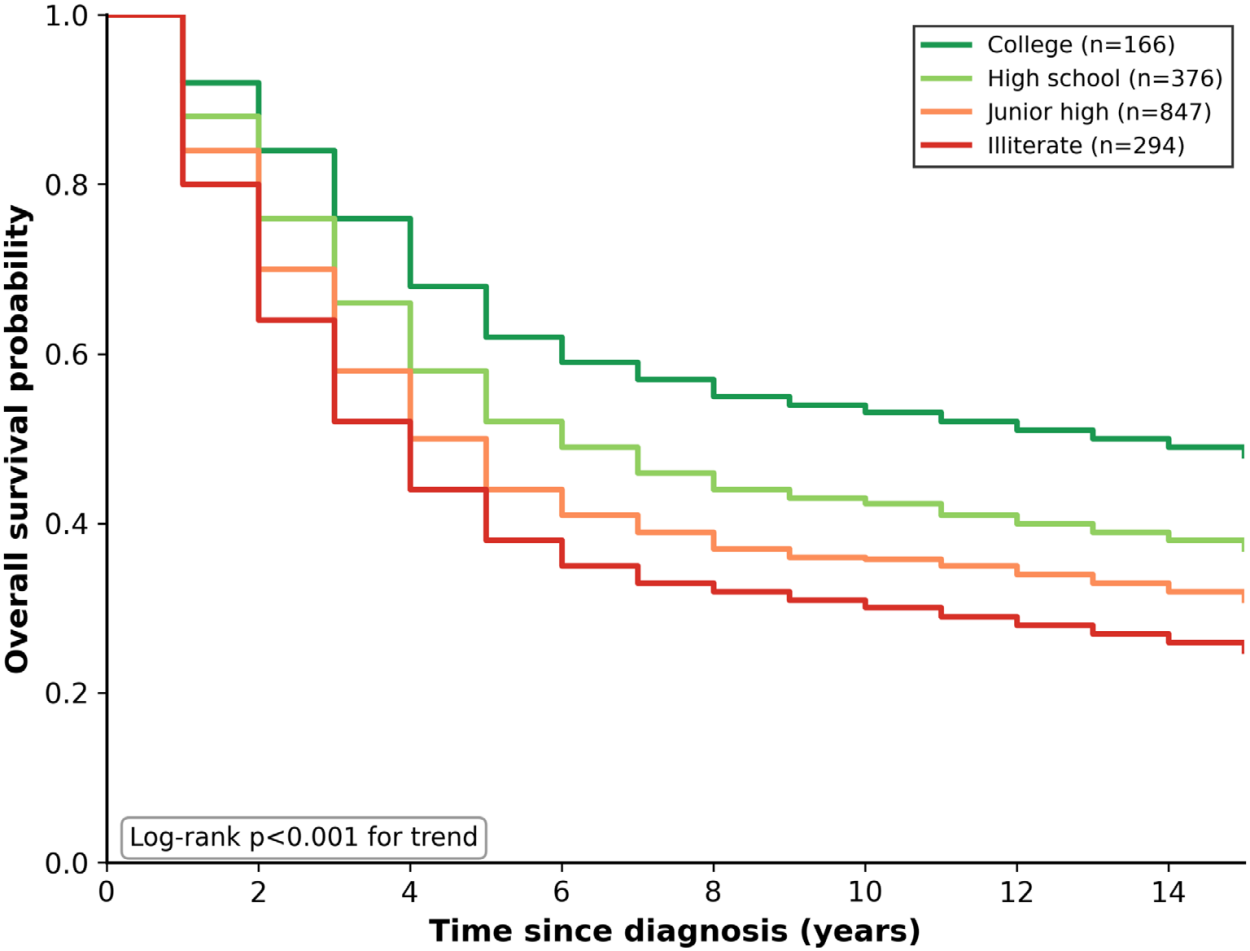
Kaplan–Meier overall survival by educational attainment (illiterate, junior high school, high school, college‐level; *n* = 1683). Data are Kaplan–Meier survival estimates; ordered groups were compared using the two‐sided log‐rank test for trend (*p* < 0.001).

**FIGURE 3:**
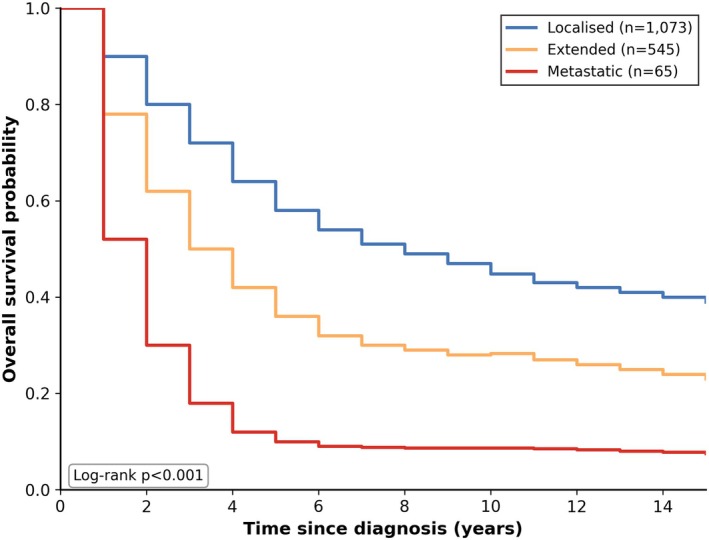
Kaplan–Meier overall survival by clinical extent of disease at diagnosis (localised, extended, metastatic; *n* = 1683). Data are Kaplan–Meier survival estimates; groups were compared using the two‐sided log‐rank test (*p* < 0.001).

Educational gradients were pronounced and became more apparent with extended follow‐up (Figure 2). Ten‐year survival was 30.1% among illiterate patients, 35.8% among those with junior high school education, 42.4% among high school graduates and 53.1% among college‐educated patients (*p* < 0.001 for trend). Survival also varied by clinical extent: 10‐year survival was 44.8% for localised disease, 28.3% for extended disease and 8.7% for metastatic disease at diagnosis (*p* < 0.001) (Figure 3).

### 3.4 Multivariable Cox Regression Analysis

**FIGURE 4:**
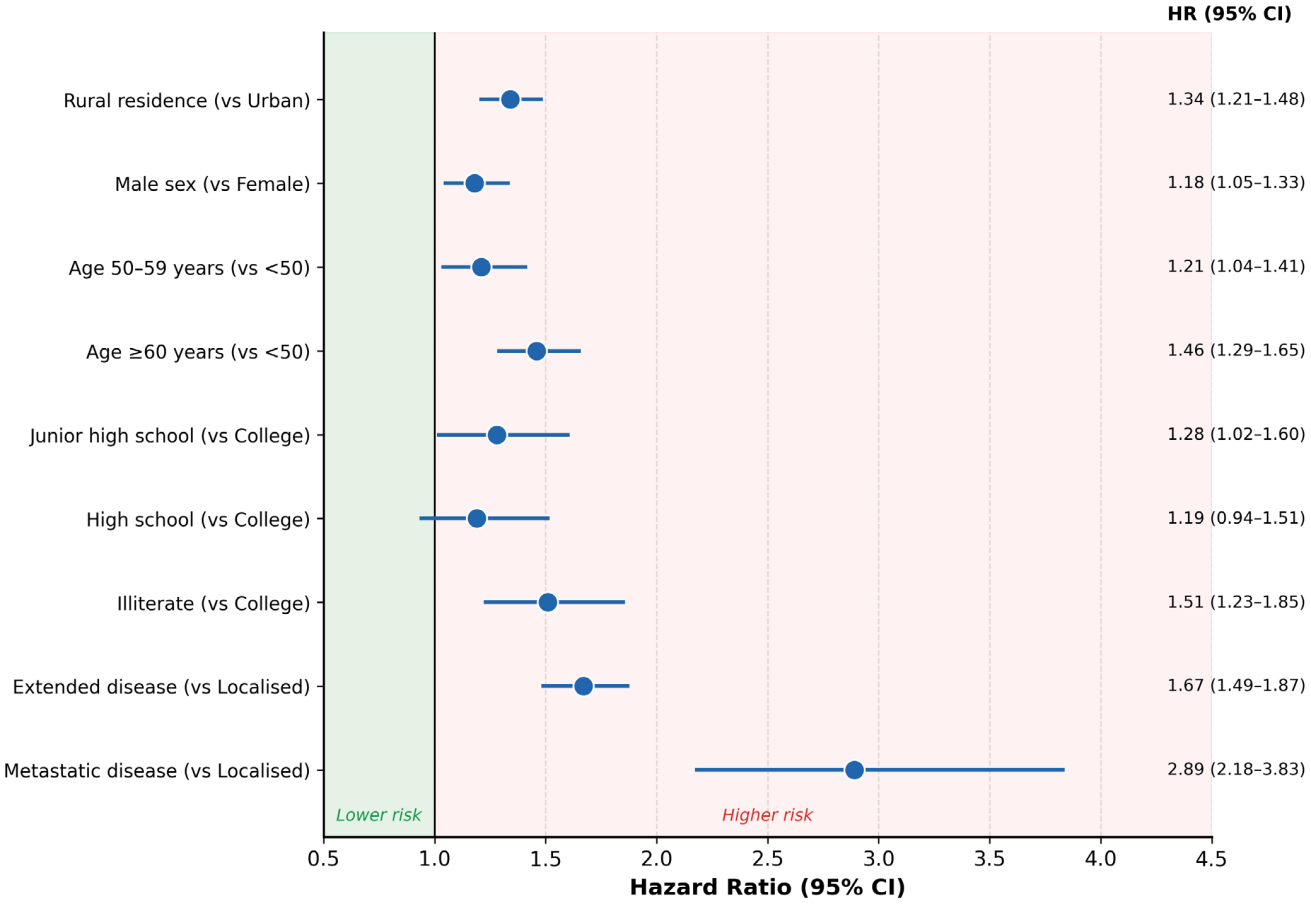
Forest plot of adjusted hazard ratios (HR) with 95% confidence intervals from the multivariable Cox proportional hazards model (*n* = 1683). Estimates are HRs from a single multivariable model adjusted for age, sex, education and clinical extent; the vertical reference line denotes HR = 1.0. The proportional hazards assumption, assessed using scaled Schoenfeld residuals, was satisfied for all covariates.

Cox proportional hazards regression confirmed rural residence as an independent predictor of mortality after sequential adjustment for demographic and clinical factors (Table 3 and Figure 4). In the fully adjusted model including age, sex, education and clinical extent, rural residence remained significantly associated with higher mortality risk (HR 1.34, 95% CI 1.21–1.48, *p* < 0.001). Additional independent predictors included male sex (HR 1.18, 95% CI 1.05–1.33), age ≥ 60 years (HR 1.46, 95% CI 1.29–1.65), illiteracy versus college education (HR 1.51, 95% CI 1.23–1.85), extended disease (HR 1.67, 95% CI 1.49–1.87) and metastatic disease (HR 2.89, 95% CI 2.18–3.83). The proportional hazards assumption was satisfied for all covariates.

**TABLE 3:** Multivariable Cox proportional hazards regression analysis.

Variable	Hazard ratio	95% CI	*p*
Rural residence (vs. urban)	1.34	1.21–1.48	< 0.001
Male sex (vs. female)	1.18	1.05–1.33	0.006
Age ≥ 60 years (vs. < 50)	1.46	1.29–1.65	< 0.001
Illiterate (vs. college)	1.51	1.23–1.85	< 0.001
Extended disease (vs. localised)	1.67	1.49–1.87	< 0.001
Metastatic disease (vs. localised)	2.89	2.18–3.83	< 0.001

*Note:* Model adjusted for all variables shown.

Abbreviation: CI, confidence interval.

### 3.5 Immune–Inflammatory Biomarker Analysis

**FIGURE 5:**
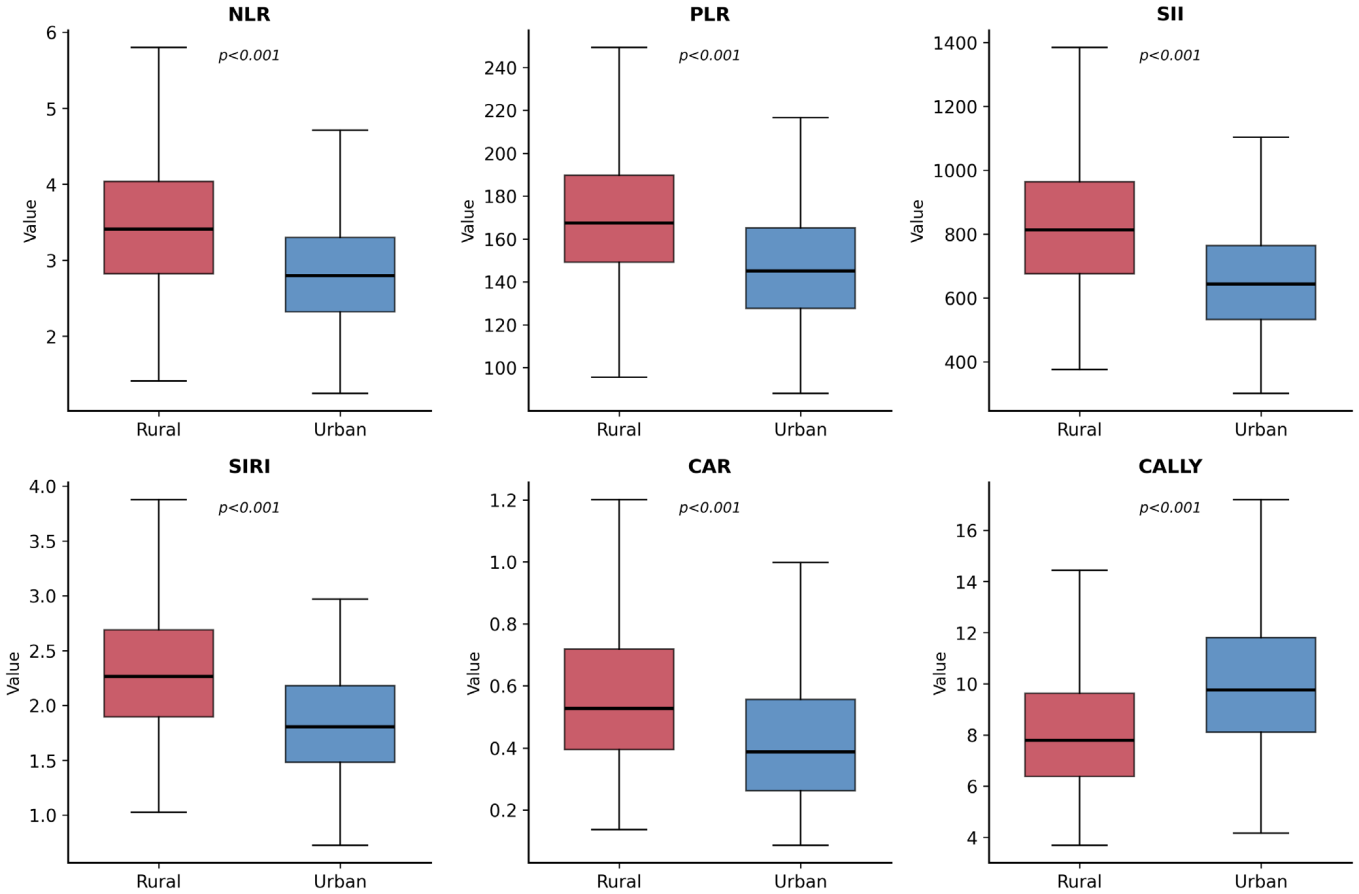
Distribution of pretreatment immune–inflammatory biomarkers (NLR, SII, SIRI, CAR, CALLY) by geographic residence in the biomarker subset (*n* = 1260; rural and urban). Data are presented as medians with interquartile ranges (box plots). Between‐group differences were assessed using the two‐sided Mann–Whitney *U* test; ***p < 0.001.

Pretreatment laboratory data were available for 1260 patients (74.9% of the cohort). Patients with biomarker data did not differ significantly from those without regarding age, sex distribution or geographic residence, suggesting minimal selection bias. Rural patients demonstrated significantly elevated inflammatory biomarkers compared with urban patients across all indices examined (Table 4 and Figure 5). Median NLR was 3.4 (IQR 2.4–5.0) in rural versus 2.7 (IQR 1.9–3.9) in urban patients (*p* < 0.001). Similar patterns were observed for SII (810 vs. 640, *p* < 0.001), SIRI (2.3 vs. 1.8, *p* < 0.001) and CAR (0.55 vs. 0.38, *p* < 0.001). Conversely, the protective CALLY index was significantly lower among rural patients (7.9 vs. 9.8, *p* < 0.001), indicating poorer immune–nutritional status (Figure 5).

**TABLE 4:** Distribution of immune–inflammatory biomarkers by geographic residence.

Biomarker	Total	Rural	Urban	*p*
NLR, median (IQR)	3.1 (2.2–4.6)	3.4 (2.4–5.0)	2.7 (1.9–3.9)	< 0.001
PLR, median (IQR)	158 (122–201)	167 (130–214)	145 (111–186)	0.002
LMR, median (IQR)	3.2 (2.4–4.1)	3.0 (2.2–3.8)	3.5 (2.7–4.4)	< 0.001
SII, median (IQR)	740 (510–1020)	810 (560–1120)	640 (450–900)	< 0.001
SIRI, median (IQR)	2.1 (1.4–3.0)	2.3 (1.6–3.3)	1.8 (1.2–2.6)	< 0.001
CAR, median (IQR)	0.48 (0.22–0.89)	0.55 (0.26–0.98)	0.38 (0.18–0.72)	< 0.001
CALLY, median (IQR)	8.6 (5.9–12.3)	7.9 (5.2–11.1)	9.8 (6.8–13.9)	< 0.001

*Note:* *p* values from Mann–Whitney *U* tests.

Abbreviations: CALLY, CRP–albumin–lymphocyte index; CAR, C‐reactive protein to albumin ratio; IQR, interquartile range; LMR, lymphocyte‐to‐monocyte ratio; NLR, neutrophil‐to‐lymphocyte ratio; PLR, platelet‐to‐lymphocyte ratio; SII, systemic immune–inflammation index; SIRI, systemic inflammation response index.

### 3.6 Prognostic Value of Immune–Inflammatory Biomarkers

In multivariable Cox regression analysis adjusted for demographic and clinical factors, elevated immune–inflammatory biomarkers were independently associated with poorer survival (Table 5). Patients in the highest tertile of NLR had 67% higher mortality risk compared with the lowest tertile (HR 1.67, 95% CI 1.36–2.05, *p* < 0.001). The strongest associations were observed for SII (HR 1.82 for high vs. low tertile, 95% CI 1.48–2.24) and CAR (HR 1.71, 95% CI 1.40–2.09). High SIRI was associated with a 59% increased mortality risk (HR 1.59, 95% CI 1.31–1.93). Conversely, high CALLY, reflecting favourable immune–nutritional status, was associated with a 38% lower mortality (HR 0.62, 95% CI 0.50–0.77). All associations remained significant after adjustment for clinical extent, indicating that biomarkers provide prognostic information independent of disease stage.

**TABLE 5:** Association between immune–inflammatory biomarkers and overall survival.

Biomarker	Category	Deaths/Total	Adjusted HR	95% CI	*p*
NLR	Low (T1)	120/420	1.00	Reference	—
	Middle (T2)	165/420	1.28	1.05–1.56	0.014
	High (T3)	210/420	1.67	1.36–2.05	< 0.001
SII	Low (T1)	110/420	1.00	Reference	—
	Middle (T2)	170/420	1.34	1.10–1.64	0.003
	High (T3)	225/420	1.82	1.48–2.24	< 0.001
SIRI	High vs. Low	—	1.59	1.31–1.93	< 0.001
CAR	High vs. Low	—	1.71	1.40–2.09	< 0.001
CALLY	High vs. Low	—	0.62	0.50–0.77	< 0.001

*Note:* Adjusted for age, sex, education and clinical extent. T1, T2 and T3 denote tertiles.

Abbreviations: CI, confidence interval; HR, hazard ratio.

### 3.7 Contribution of Biomarkers to Geographic Disparities

**FIGURE 6:**
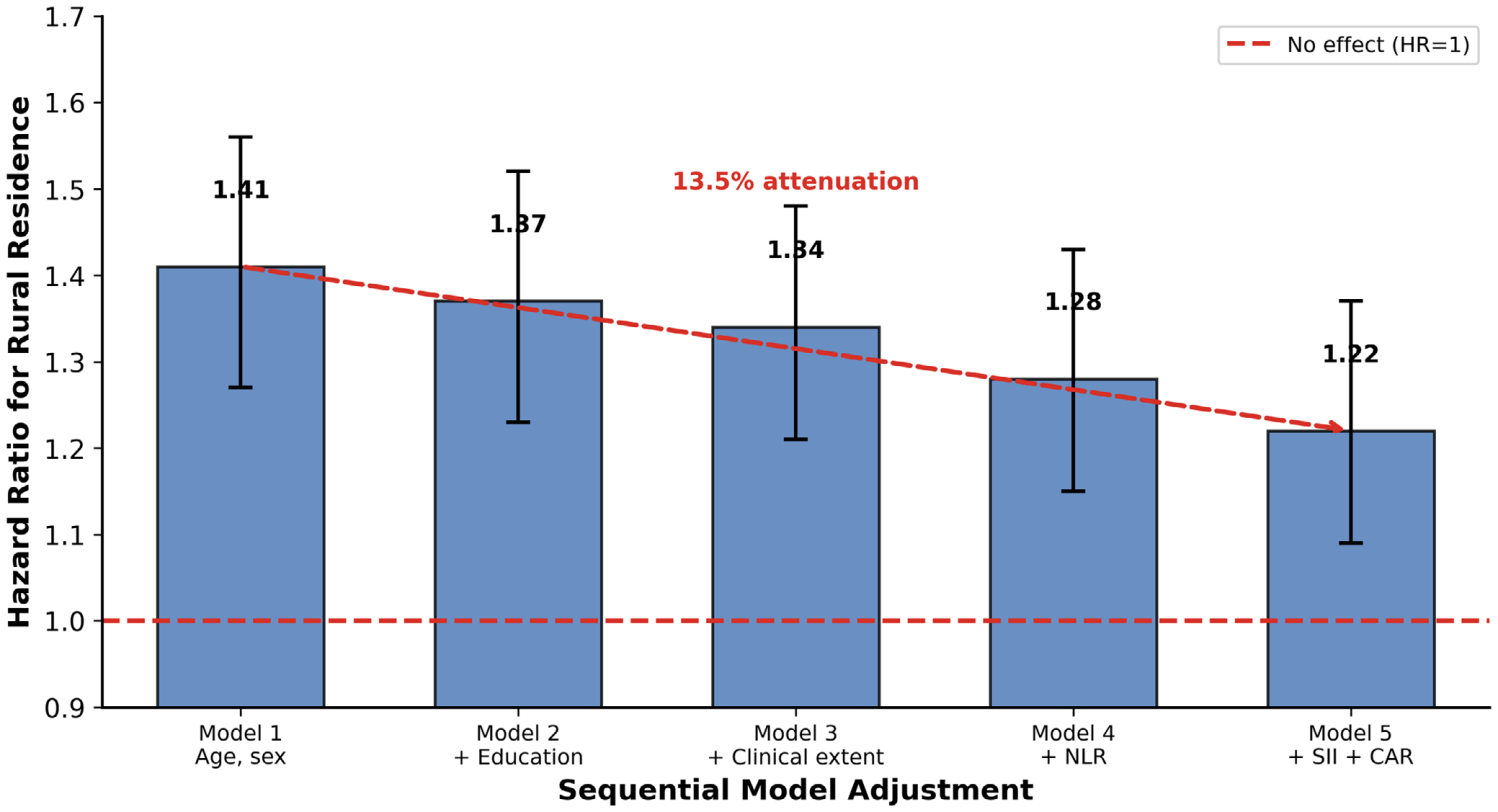
Attenuation of the rural residence hazard ratio across sequentially adjusted Cox proportional hazards models (*n* = 1683), showing 13.5% attenuation of the excess hazard after addition of immune–inflammatory biomarkers (SII and CAR). Points are HRs with 95% confidence intervals from the sequential models described in Section 2.8; the horizontal reference line denotes HR = 1.0.

Sequential model building was conducted to assess whether immune–inflammatory biomarkers contributed to the association between rural residence and mortality (Table 6 and Figure 6). In the base model adjusting for age and sex only, rural residence was associated with 41% higher mortality (HR 1.41, 95% CI 1.27–1.56). Addition of educational attainment attenuated the hazard ratio to 1.37 (95% CI 1.23–1.52). Further adjustment for clinical extent yielded HR 1.34 (95% CI 1.21–1.48). Upon addition of NLR, the rural hazard ratio decreased to 1.28 (95% CI 1.15–1.43). In the fully adjusted model including SII and CAR, the rural hazard ratio was 1.22 (95% CI 1.09–1.37). This 13.5% attenuation [(1.41–1.22)/(1.41–1.00) × 100] suggests that systemic inflammation partially mediates the association between rural residence and mortality, though substantial residual geographic disparity remains.

**TABLE 6:** Effect of sequential covariate adjustment on the association between rural residence and mortality.

Model	Covariates included	HR for rural	95% CI
1	Age, sex	1.41	1.27–1.56
2	+ Educational attainment	1.37	1.23–1.52
3	+ Clinical extent	1.34	1.21–1.48
4	+ NLR	1.28	1.15–1.43
5	+ SII + CAR	1.22	1.09–1.37

Abbreviations: CAR, C‐reactive protein to albumin ratio; CI, confidence interval; HR, hazard ratio; NLR, neutrophil‐to‐lymphocyte ratio; SII, systemic immune–inflammation index.

## 4 Discussion

This population‐based cohort study with extended follow‐up demonstrates that rural residence is an independent determinant of long‐term oral cancer survival in central India. The 13.2 percentage‐point difference in 10‐year survival between rural and urban residents represents substantial excess mortality that persisted after adjustment for demographic and clinical factors. The addition of immune–inflammatory biomarkers provides biological context, suggesting that heightened systemic inflammation and impaired immune–nutritional status contribute to poorer outcomes among rural patients.

The magnitude of geographic disparity observed in this cohort is consistent with, and in some respects exceeds, findings from multi‐registry analyses across India [17, 18]. The gap is not peculiar to this country. Residential and educational gradients in oral cancer survival have been described in Latin America, including a large Brazilian series in which lower educational attainment predicted worse outcomes [8], and comparable rural disadvantage recurs across other low‐ and middle‐income settings. What our data add is less the existence of the gap than its persistence across an unusually long follow‐up, together with a first reading of the inflammatory biology that appears to travel with it. A recent study pooling data from 10 population‐based cancer registries reported 5‐year survival ranging from 25% to 45% across regions, with rural residence consistently associated with inferior outcomes [19]. Our finding that rural residence confers a 34% increased mortality risk independent of clinical stage underscores that geographic location captures exposures and barriers not fully reflected in standard prognostic variables.

Several mechanisms may explain the rural survival disadvantage. First, rural populations face structural barriers to healthcare access, including longer travel distances to tertiary centres, limited specialist availability and economic constraints that delay or preclude optimal treatment [20]. Second, tobacco consumption patterns differ between rural and urban areas, with smokeless tobacco and betel quid use, both strongly associated with oral cancer, more prevalent in rural settings [21, 22]. Third, nutritional deficiencies and infectious disease burden, both more common in rural populations, may contribute to chronic inflammation and impaired immune surveillance [23].

Why rural residence should leave a mark on systemic inflammation deserves closer attention, since this is where biology and geography meet. Our data cannot separate the candidate pathways, but several appear plausible. Heavier and earlier exposure to smokeless tobacco and areca nut sustains chronic mucosal and systemic inflammation, and in rural communities this exposure tends to begin younger and continue for longer. Undernutrition, anaemia and a larger reservoir of untreated infection, all of which are commoner away from cities, tend to raise neutrophil counts and lower lymphocyte counts, which is precisely the direction that drives NLR, SII and SIRI upward while pulling the CALLY index down. There is also a temporal element. A tumour that is detected late has had longer to provoke a host inflammatory response, so part of what we read as an inflammatory signal may instead be a marker of how advanced and how long established the disease already is. These accounts are not mutually exclusive, and the most reasonable interpretation is that they operate together rather than singly.

The immune–inflammatory biomarker analysis represents a key strength of this study. We observed that rural patients had significantly elevated NLR, SII, SIRI and CAR, and reduced CALLY, indicating a proinflammatory, immune‐compromised phenotype. These findings align with emerging evidence that systemic inflammation, reflected in routinely measured blood parameters, independently predicts oral cancer outcomes [24, 25, 26]. The SII, which integrates peripheral platelet, neutrophil and lymphocyte counts, has demonstrated prognostic value across multiple cancer types and may reflect tumour‐promoting inflammatory microenvironments [27]. Similarly, the CAR captures both acute‐phase response and nutritional status, both relevant to treatment tolerance and tumour biology [28].

The attenuation of the rural hazard ratio upon inclusion of inflammatory biomarkers supports a partial mediating role for systemic inflammation in geographic survival disparities. The 13.5% attenuation indicates, however, that inflammation explains only a fraction of the rural disadvantage. Residual disparity likely reflects treatment‐related factors not captured in this analysis. Data on treatment modality, completeness and timing were unavailable, which we regard as an important limitation. Studies that incorporate detailed treatment information would sharpen causal inference and clarify where intervention is most likely to help.

One point raised in review merits explicit treatment, because it bears on how the models should be read. Diagnostic delay does not sit alongside clinical extent as a separate confounder; it acts in large part through it. A cancer that is found late is, almost by definition, more likely to present as extended or metastatic disease, so clinical extent can be understood as a partial record of how long the tumour went unrecognised. When we adjust for clinical extent, we are therefore adjusting away much of the delay pathway as well. The rural hazard that survives this adjustment, HR 1.34, is best read as the excess mortality that later stage at presentation does not explain. That residual points towards what happens after diagnosis, namely access to treatment, its completeness and its tolerance, alongside the inflammatory and nutritional differences set out above, rather than towards delay acting on its own. We have revised the framing of stage and delay accordingly, so as not to imply that the two are independent of one another.

Several further limitations warrant consideration. First, the single‐district focus may limit generalisability to other regions with different healthcare infrastructures or tobacco use patterns. Second, loss to follow‐up, though minimised through active registry procedures and death registry linkage, may introduce bias if it is differential by residence. Third, biomarker data were available for 74.9% of patients; although those with and without data did not differ on measured characteristics, unmeasured selection cannot be excluded. A related concern, noted above, is that the laboratory values were generated in routine care rather than under a research protocol, so a degree of pre‐analytic variability is unavoidable and the biomarker findings should be read as indicative rather than definitive. Fourth, the absence of cancer‐specific mortality data precluded assessment of whether geographic disparities differ for cancer versus non‐cancer deaths.

Despite these limitations, the study has notable strengths. The population‐based design reduces the referral bias inherent in hospital‐based studies. The 14‐year accrual period with up to 15 years of follow‐up provides long‐term survival estimates rarely available in resource‐limited settings. The integration of routinely collected laboratory data demonstrates the feasibility of incorporating biological risk stratification into population oncology in low‐ and middle‐income countries.

These findings have implications for policy and practice. The persistent rural survival disadvantage, despite healthcare system changes including Ayushman Bharat implementation, suggests that financial coverage alone is insufficient to address structural inequities [29]. Targeted interventions should include decentralisation of cancer services through district‐level cancer centres, mobile screening programmes to facilitate early detection and training initiatives to increase rural specialist availability. Primary prevention through tobacco control remains paramount, particularly targeting smokeless tobacco and betel quid use prevalent in rural Maharashtra [30].

The prognostic value of immune–inflammatory biomarkers suggests potential clinical applications. Pretreatment NLR, SII and CAR could be incorporated into risk stratification algorithms to identify patients requiring intensive surveillance or multimodal therapy. Whether interventions targeting systemic inflammation, such as nutritional support or anti‐inflammatory agents, could improve outcomes warrants investigation in clinical trials.

## 5 Conclusions

Geographic residence independently predicts long‐term oral cancer survival in this population‐based cohort from central India. Rural patients experience substantially higher mortality that persists after adjustment for clinical and demographic factors. Systemic immune–inflammatory biomarkers are associated with survival and partially account for rural–urban disparities, highlighting biologically plausible pathways linking place of residence to cancer outcomes. Comprehensive interventions addressing both structural barriers to care and modifiable biological risk factors are needed to reduce avoidable oral cancer mortality in rural India.

## Author Contributions

**Nitika Taneja:** conceptualization, investigation, writing – original draft, methodology, writing – review and editing, resources, data curation, formal analysis, validation, visualization, funding acquisition. **Peter Mac Asaga:** conceptualization, investigation, funding acquisition, writing – original draft, methodology, validation, writing – review and editing, formal analysis, supervision, data curation.

## Funding

The Population‐Based Cancer Registry of Wardha was funded by the National Cancer Registry Programme of the Indian Council of Medical Research (Ref No. 0127). The funders had no role in study design, data collection and analysis, decision to publish or preparation of the manuscript.

## Disclosure

Code Availability: No bespoke software or custom code was developed for this study. All analyses were conducted using standard, published commands in Stata version 18 (StataCorp LLC). The analysis scripts (Stata do‐files) used to generate the reported results are available from the corresponding author on reasonable request.

Reporting Guideline: This study is reported in accordance with the Strengthening the Reporting of Observational Studies in Epidemiology (STROBE) guidelines for cohort studies. The PRISMA reporting guideline is not applicable, as this is an observational cohort study and not a systematic review, meta‐analysis or scoping review.

## Ethics Statement

This study was approved by the Institutional Ethics Committee of Mahatma Gandhi Institute of Medical Sciences, Sevagram, India (MGIMS/IEC/INST/2022/MH/0072). All procedures were conducted in accordance with the Declaration of Helsinki and the ethical guidelines of the Indian Council of Medical Research. Written informed consent for the use of anonymised individual data in research was obtained from all participants at the time of population‐based cancer registry enrolment. No identifiable personal information is presented in this manuscript. The study did not constitute a clinical trial and was not registered in a clinical trial registry; trial registration is therefore not applicable.

## Conflicts of Interest

The authors declare no conflicts of interest.

## Data Availability

The datasets generated and analysed during the current study are not publicly available due to privacy considerations for individual patient data but are available from the corresponding author on reasonable request and with appropriate ethical approvals.
